# Automating the creation of fashion patterns using deep learning algorithms

**DOI:** 10.3389/frai.2026.1828627

**Published:** 2026-08-19

**Authors:** Randa Alsabhi

**Affiliations:** Fashion Design and Textile Department, University of Tabuk, Tabuk, Saudi Arabia

**Keywords:** artificial intelligence, deep learning, fashion pattern generation, computer-aided design, multimodal learning, Vision Transformer, Generative Adversarial Network, digital fashion

## Abstract

**Introduction:**

Fashion pattern generation remains one of the most labor-intensive stages in garment production because it depends heavily on expert manual drafting, iterative revisions, and technical precision. Although existing artificial intelligence (AI)-based fashion systems have primarily focused on garment classification, image synthesis, and trend prediction, limited research has addressed the automated generation of structured garment pattern representations incorporating manufacturing-oriented construction information. This study addresses this gap by developing an end-to-end deep learning framework for automated fashion pattern generation.

**Methods:**

The proposed framework converts garment images, sketches, and textual construction descriptions into structured, CAD-compatible fashion pattern representations. It integrates Vision Transformer (ViT)-based visual feature extraction, BERT-based textual encoding, multimodal feature fusion, and an encoder–decoder Generative Adversarial Network (GAN) for iterative pattern generation and refinement. Automated seam and notch generation modules are additionally incorporated to enhance manufacturing compatibility and CAD integration. The framework was experimentally evaluated using the DeepFashion dataset and compared with CNN and conditional GAN baseline models under identical experimental conditions.

**Results:**

The proposed framework demonstrated strong performance, achieving an Intersection over Union (IoU) score of 0.93, an average landmark alignment error of 3.2 pixels, and an aesthetic consistency score of 9.5/10. These results outperformed the CNN and conditional GAN baseline models across the evaluated performance measures.

**Discussion:**

The findings demonstrate the potential of the proposed multimodal deep learning framework to provide a scalable and production-oriented solution for automated fashion pattern generation. By integrating visual, textual, and manufacturing-oriented information, the framework can reduce pattern development time, improve pattern consistency, and support mass customization within digital fashion production workflows.

## 1 Introduction

The fashion industry, prized for its creativity and craftsmanship, is now undergoing a radical transformation due to increasingly adopting advanced technologies promising the transformation of traditional workflows (Abdel Halim et al., 2024). Being one of the biggest, fastest-moving industries globally, it finds itself facing relentless stress for innovation in cutting down production timelines and responding effectively to the fast-changing expectations of its global, diverse clientele (Centobelli et al., 2022). One of the strong enablers has been the challenge of integrating state-of-the-art technologies, particularly Artificial Intelligence and Deep Learning, which make automation, personalization, and operational efficiencies possible at a completely different level; hence, they are pushing the boundaries in fashion design and production (Mohiuddin Babu et al., 2024).

Deep learning is a fine domain of Artificial Intelligence, which has been outstandingly providing efficient automation of complicated, usually manually performed tasks in several fields (Mosqueira-Rey et al., 2023). It performs excellently on large volumes of data, finding complex patterns and executing a task with near-human-level performance. Some of the key applications that involve the generation of images are driven by Deep Learning models, including photorealistic visuals generated through creative algorithms, such as Generative Adversarial Networks (GANs), object recognition with a greater enhancement of precise identification in images by various key technologies involving autonomous vehicles, medical diagnosis, among so many others; while Natural Language Processing (NLP) is one area that is really making toward intelligent virtual assistants and advanced text analytics (Shahriar, 2022). All these acts of success underpin the potential of deep learning in transforming industries that rely heavily on creativity and pattern recognition, hence becoming highly relevant within the fashion sector.

Deep learning has started to be adopted in the fashion world to classify garments, generate fabric patterns, and predict trends (Imtiaz et al., 2024). One of the most labor- and complicated-intensive processes in the whole flow is the creation of the fashion pattern itself, which is rarely explored. Patternmaking in fashion is the backbone of garment production, translating the concept into a technically specific pattern with great detail on how the fabric will be cut, shaped, and assembled into a product (Lei and Li, 2021). The requirement for this would be a very high level of expertise, precision, and creativity, possibly repeated in various revisions as needed, to actualize the exact intentions of the designer. This traditionally constitutes a bottleneck in the production pipeline, bounding the speed and scalability of design workflows.

The lack of Deep Learning applications for patternmaking automation opens opportunities for solving the yet-unresolved series of problems in the fashion industry. Automation of fashion patterns could significantly reduce the need for human expertise and make the design process more available to unqualified persons, thus democratizing creativity. This could also drastically cut down production time from conceptualization to production. In addition, automation could allow for more innovative outputs with new and unique patterns not thought of by the human mind, therefore extending the frontier of traditional design. This will also have the potential for effective scaling of operations, hence supporting mass customization and on-demand manufacturing, which is an increasing requirement within today’s fast-paced consumer-driven markets.

Despite such advantages, there are indeed challenges to be encountered in the automation of fashion pattern creation. Such a system must maintain design fidelity-what comes out should represent the designer’s ideas. It has to be able to capture the complexity, including intricate designs for varied garment types-from casual wear to high fashion. The system needs to balance creativity with practicality: it has to generate novel yet manufacturable patterns using existing technologies. This study is being conducted to overcome these challenges through the integration of Deep Learning models in the automation of fashion patternmaking processes. The focus will be to create an end-to-end automation to have a strong framework for incorporating Deep Learning into fashion design workflows. It combines new neural network architectures, including encoder–decoder models and GANs, where the designs become highly accurate and CAD-compatible pattern representations from input sketches, digital illustrations, or any other form. The contribution of the research study to extensive experimentation and its evaluation will establish the viability or advantages of the approach, hence adding to the ever-growing knowledge base of Artificial Intelligence applications within the creative industries. It fills an extremely important gap in the existing literature; therefore, it could prove a scalable and innovative solution to one of the most manually intensive processes in fashion. The results also demonstrate broad applicability, from fashion design and manufacturing to other design-based industries, further cementing the role Artificial Intelligence and Deep Learning may be able to play in fostering creativity, efficiency, and innovation.

## 2 Related work

### 2.1 Deep learning approaches in fashion pattern and garment design

Automated fashion design has become an increasingly important research area due to the growing demand for efficient design workflows, personalization, and scalable garment production. Recent advances in Deep Learning have significantly contributed to the development of intelligent systems capable of generating visually appealing and functionally relevant fashion designs. Among these approaches, Generative Adversarial Networks (GANs) have demonstrated strong capabilities in generating novel garment patterns and fashion concepts from large-scale visual datasets (Yan et al., 2023). GAN-based architectures employ a generator–discriminator framework that enables the synthesis of realistic garment structures and stylistic variations. Despite their effectiveness in image generation, many existing GAN-based fashion systems focus primarily on visual realism rather than manufacturable pattern generation or production compatibility.

Research has also explored personalized fashion generation approaches that incorporate user-specific information such as body scans, garment preferences, and style characteristics (Jiang et al., 2021). These methods aim to enhance user satisfaction and customization capabilities by adapting generated outputs to individual requirements. However, most personalization-oriented systems remain focused on recommendation or visualization tasks and provide limited support for technically accurate garment construction or CAD-compatible pattern outputs.

Style transfer techniques have further contributed to fashion pattern generation by enabling the integration of multiple stylistic features into a unified garment representation (Zheng et al., 2024). Such approaches allow designers to experiment with hybrid esthetics while preserving the structural characteristics of the original design. Nevertheless, style transfer methods often prioritize visual creativity over structural manufacturability and may generate patterns that are esthetically appealing but difficult to implement in real garment production workflows.

Recent developments in multi-modal Deep Learning have introduced systems capable of integrating images, sketches, and textual garment descriptions to improve design understanding and generation quality (Liu et al., 2021). Multi-modal architectures enhance semantic interpretation by combining complementary information from different data sources, thereby enabling richer and more context-aware pattern generation. However, many existing multi-modal approaches lack precise structural alignment mechanisms required for technical fashion pattern construction and industrial manufacturing applications.

Conditional GAN (cGAN)-based methods have also been proposed to improve control over generated fashion outputs by introducing design constraints such as color schemes, garment categories, and geometric configurations (Liu and Zhou, 2022). These methods provide designers with greater influence over the generation process and improve output consistency. Despite these improvements, existing conditional generation approaches still face challenges related to seam alignment, landmark precision, manufacturability validation, and integration with industrial CAD systems.

### 2.2 Machine learning applications in related design domains

In addition to direct fashion design applications, several studies have explored machine learning techniques in related design and material-oriented domains. Reinforcement learning approaches have been utilized to optimize garment comfort, structural adaptability, and visual appeal through reward-driven pattern refinement processes (Jangir et al., 2020). These methods demonstrate the potential of iterative learning mechanisms for improving design optimization and adaptive garment behavior. Although such approaches are not directly focused on technical fashion pattern generation, their optimization strategies provide valuable insights into automated refinement and structural adjustment processes relevant to garment manufacturing.

Explainability and interpretability in Artificial Intelligence systems have also received growing attention in design-oriented applications (Moosavi et al., 2020). Researchers have developed visualization and interpretation tools that help designers better understand Deep Learning model decisions and generated outputs. Such explainable AI techniques are particularly important in creative industries where designer trust, transparency, and controllability are critical factors. Although these studies originate from broader AI application domains, their methodologies are highly relevant for improving transparency and user confidence in automated fashion pattern generation systems.

### 2.3 Recent advances in diffusion models and digital garment generation

More recently, diffusion models have emerged as a powerful alternative to Generative Adversarial Networks for fashion synthesis and digital garment generation. Unlike GANs, which learn garment distributions through adversarial competition between generator and discriminator networks, diffusion models progressively learn to reverse a noise-corruption process, enabling high-fidelity and controllable generation. Their ability to preserve fine visual details and incorporate multiple conditioning signals has led to increasing adoption in virtual try-on, garment appearance synthesis, texture generation, and fashion design applications. Recent diffusion-based virtual try-on methods, for example, have demonstrated improved preservation of garment textures and structural details while reducing some of the deformation and visual artifacts associated with earlier GAN-based approaches (Morelli et al., 2023; Choi et al., 2024).

Beyond virtual try-on, diffusion models are increasingly being extended toward three-dimensional digital garment creation. Garment3DGen introduced an image-guided framework for generating simulation-ready 3D garment assets by combining diffusion-based image-to-3D generation with mesh deformation and texture synthesis (Sarafianos et al., 2025). Similarly, FabricDiffusion demonstrated the use of denoising diffusion models for recovering distortion-free garment textures from clothing images and transferring these textures to 3D garments (Zhang et al., 2024). These developments indicate a broader transition from purely image-based fashion generation toward digital garment representations that can support downstream simulation and interactive design applications.

Parallel advances have focused directly on the relationship between garment appearance, three-dimensional geometry, and sewing-pattern representations. Panelformer proposed a transformer-based framework for reconstructing complete sewing patterns from two-dimensional garment images by predicting individual panels and their stitching relationships (Chen et al., 2024). GarmentCodeData further contributed a large-scale dataset containing 115,000 made-to-measure 3D garments together with corresponding sewing patterns, body variations, and material configurations, providing an important resource for learning-based garment generation and reconstruction (Korosteleva et al., 2024). More recently, AIpparel introduced a multimodal foundation model trained on more than 120,000 garments with text, image, and sewing-pattern information, enabling text-to-garment, image-to-garment, and interactive sewing-pattern generation and editing (Nakayama et al., 2025). Design2GarmentCode similarly demonstrated the potential of large multimodal models to translate images, textual descriptions, sketches, or combinations of these inputs into parametric pattern-making programs and vectorized sewing patterns (Zhou et al., 2025).

Recent diffusion research has moved even closer to the objective of the present study. SewingLDM introduced a multimodal latent diffusion framework specifically for complex sewing-pattern generation conditioned on textual prompts, garment sketches, and body shapes (Liu et al., 2025). By learning sewing-pattern distributions in a compact latent representation, the approach demonstrated that diffusion architectures can provide detailed control over garment structure while accommodating different body configurations. This represents an important development beyond diffusion-based fashion visualization because the generated representation corresponds directly to garment construction rather than only to rendered garment appearance.

These advances highlight two complementary directions in contemporary AI-assisted fashion design. Diffusion models provide strong generative fidelity and flexible conditional control, whereas emerging digital-garment frameworks increasingly represent garments through structured sewing panels, stitching relationships, parametric pattern descriptions, and simulation-ready three-dimensional assets. Nevertheless, converting heterogeneous design inputs into technically accurate, manufacturable patterns while simultaneously incorporating construction details and CAD-oriented validation remains an important challenge. The present framework addresses this requirement through the integration of visual and textual feature extraction, cross-modal fusion, landmark-guided pattern generation, adversarial refinement, automated production-detail generation, and CLO3D-based validation. Future extensions of the framework could replace or complement the GAN refinement stage with latent diffusion architectures to investigate whether diffusion-based generation can further improve structural diversity, controllability, and pattern fidelity.

Despite the considerable progress achieved in AI-assisted fashion design, several important limitations remain unresolved. Most existing studies primarily focus on garment visualization, style transfer, or image synthesis rather than generating technically accurate and manufacturable garment patterns. In addition, many approaches rely exclusively on either visual or textual information without effectively integrating multi-modal design representations. Existing GAN-based methods also provide limited control over structural garment constraints, seam alignment, and production compatibility. Furthermore, only a limited number of studies present end-to-end frameworks capable of transforming conceptual fashion inputs into CAD-compatible patterns suitable for real manufacturing workflows. Therefore, the present study proposes a unified Deep Learning framework that combines multi-modal feature extraction, GAN-based pattern generation, automated seam construction, and CAD-oriented validation within a single production-oriented pipeline.

## 3 Proposed approach

This work proposes an end-to-end Deep Learning-based automation system that generates structured, CAD-compatible fashion pattern representations with high geometric accuracy. Taking input from fashion design sketches, garment images, or even textual descriptions, it translates into structured pattern representations that can be transferred to downstream CAD and virtual garment-development workflows.

**Figure 1:**
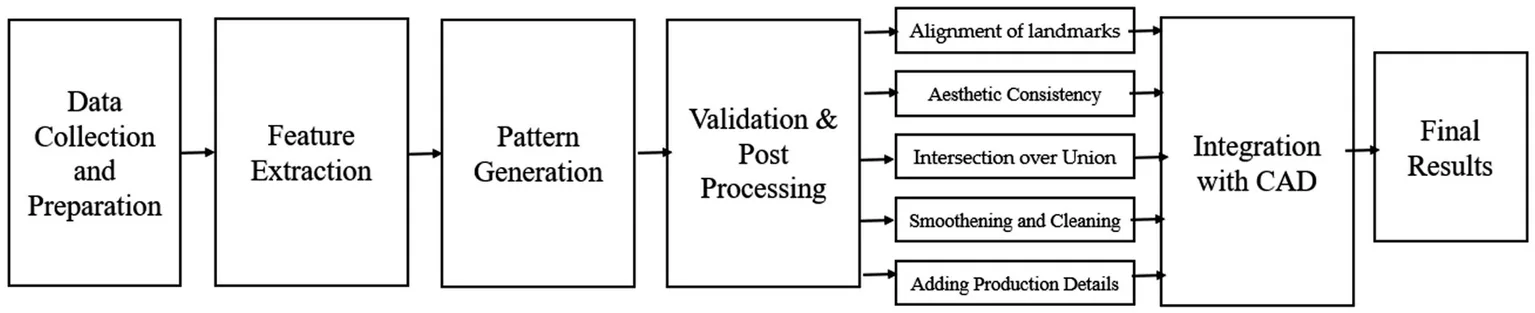
Overview of proposed approach.

Figure 1 shows the steps involved in the proposed approach.

### 3.1 Data collection and preparation

**Figure 2:**
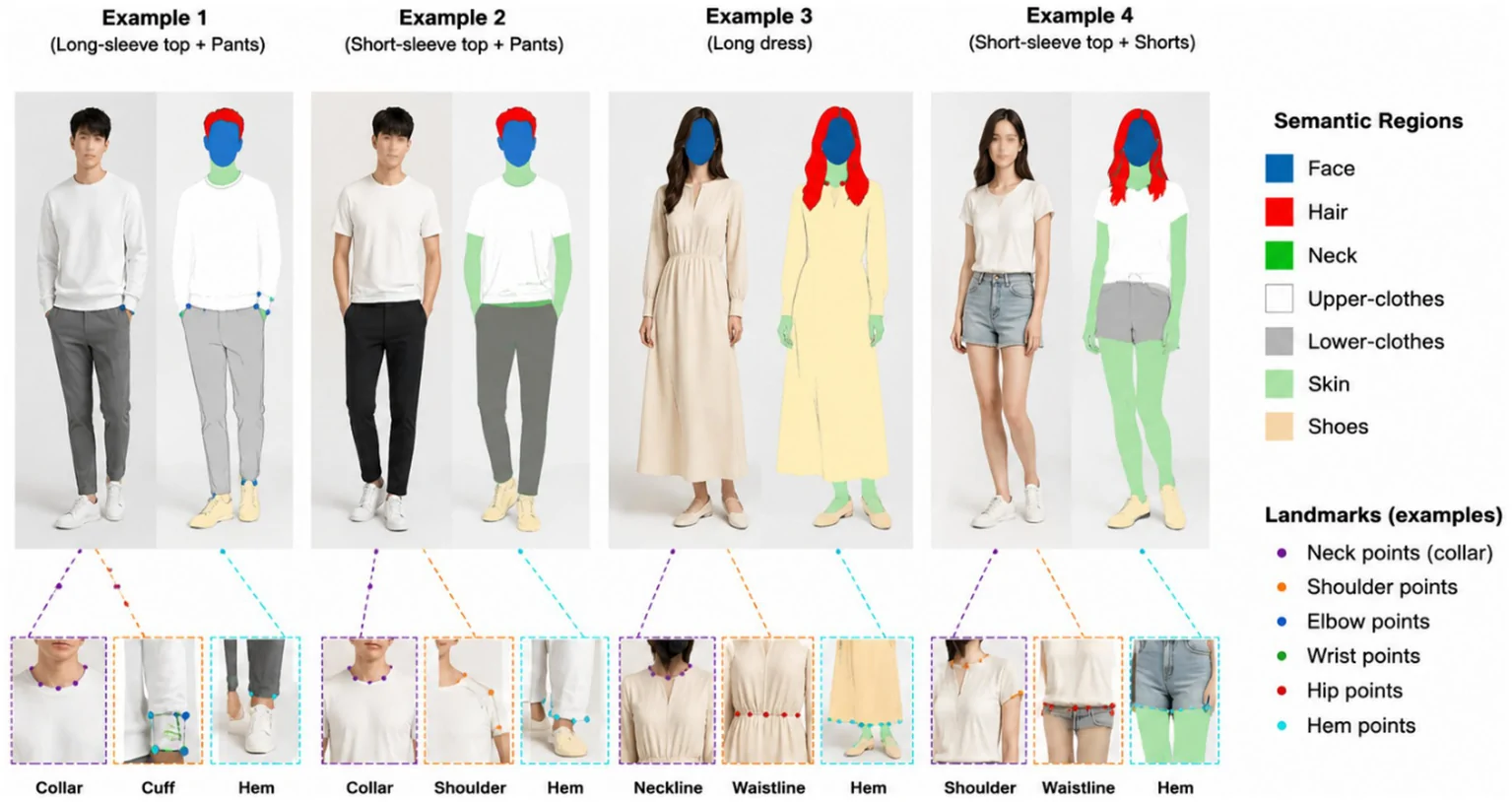
Representative examples of garment-related semantic annotations used to illustrate the visual information available in the DeepFashion dataset. Images taken from dataset: https://www.kaggle.com/datasets/vishalbsadanand/deepfashion-1/code.

This study uses the DeepFashion dataset (DeepFashion-MultiModal Dataset, n.d.), which contains more than 800,000 annotated garment images and provides information on garment categories, attributes, and structural landmarks. The dataset includes diverse garment types, such as tops, dresses, and pants, providing broad visual coverage for learning garment appearance and approximate structural characteristics. Representative annotated examples are shown in Figure 2. The available landmarks and semantic annotations provide information about visually identifiable garment regions and attributes that can support feature extraction and approximate geometric representation. However, DeepFashion does not provide professionally drafted two-dimensional sewing patterns or manufacturing annotations such as seam allowances, grainlines, notches, darts, or verified stitching relationships. Therefore, in the present study, DeepFashion is used primarily to learn garment appearance, semantic attributes, landmarks, and approximate garment geometry rather than as industrial sewing-pattern ground truth. During preprocessing, images were resized to 256 × 256 pixels and normalized, while augmentation procedures including horizontal flipping, limited rotation, and scaling were applied to the training data. DeepFashion-derived pseudo-pattern references were subsequently constructed from the available garment contours and landmark information to support model supervision and geometric evaluation. These pseudo-pattern references represent intermediate geometric approximations and should not be interpreted as professionally drafted or production-ready garment patterns.

It is important to distinguish the role of DeepFashion in the present framework from that of a technical garment-pattern dataset. DeepFashion provides garment images, category and attribute annotations, and structural landmark information, but it does not contain industrial two-dimensional sewing patterns or manufacturing annotations such as seam allowances, grainlines, notches, darts, or verified stitching relationships. Consequently, DeepFashion was used primarily for learning garment appearance, semantic attributes, structural landmarks, and approximate garment geometry rather than as ground truth for industrial patternmaking.

The pseudo-pattern representations constructed from garment contours and landmarks were therefore used as intermediate supervisory representations for model development and geometric evaluation. They should not be interpreted as verified professional pattern blocks or industrial production patterns. Production-related elements, including seam allowances, grainlines, and notches, were introduced subsequently by the proposed post-processing and construction modules rather than learned or validated against corresponding DeepFashion ground-truth annotations. Accordingly, the evaluation based on DeepFashion establishes visual and geometric consistency with the available image and landmark information, but does not independently establish industrial production readiness.

To ensure methodological consistency and reproducibility, the dataset preparation procedure followed a standardized pipeline before model training. Garment images were first screened to retain samples with valid category labels, visible garment structures, and usable landmark annotations. The retained samples were resized to 256 × 256 pixels, and pixel values were normalized before being supplied to the visual encoder. Data augmentation was applied only to the training data and included horizontal flipping, limited rotation, and scaling to increase visual variability and reduce overfitting. The prepared dataset was subsequently divided into training, validation, and testing subsets using a 70:15:15 ratio. The training subset was used for parameter optimization, the validation subset was used to monitor model performance and convergence during training, and the independent test subset was reserved for final performance evaluation.

Because DeepFashion provides garment images, attributes, categories, and landmark annotations rather than directly usable two-dimensional production patterns, pseudo-pattern reference labels were constructed for the pattern-generation task. Garment landmarks and visible boundaries were used to establish the principal structural contours, after which the extracted geometry was converted into standardized pattern representations and supplemented with construction-related elements required for model supervision. The same preprocessing and pseudo-pattern generation procedure was applied consistently across garment categories. Importantly, test samples were excluded from model optimization and augmentation-based training procedures to minimize information leakage and provide an independent assessment of generalization performance.

The experiments in this study were conducted using the publicly available DeepFashion dataset for academic research purposes. DeepFashion was originally released for non-commercial research use, and the dataset remains subject to the terms and conditions specified by its original providers. No ownership of the DeepFashion images or annotations is claimed in this study. The dataset itself is not redistributed as part of this work; researchers seeking to reproduce or extend the experiments should obtain access through the dataset’s authorized distribution source and comply with the applicable terms of use. The pseudo-pattern representations used in the present framework were derived during preprocessing from the available garment images and landmark annotations and should not be interpreted as original DeepFashion sewing-pattern annotations.

### 3.2 Feature extraction

With state-of-the-art Deep Learning models, some meaningful design elements have been extracted from the DeepFashion dataset, with visual and textual inputs embedding structural and contextual information. The former fuses the use of ViT on visual data with transformer-based text encoders, such as BERT, on textual descriptions into one unified representation for garment design (Moro et al., 2023). Visual encoding involves processing garment images and their ground-truth landmarks through the Transformer in Vision. Besides taking the traditional convolutional network route, ViT segments an image into patch pieces and treats them as sequences-like text treatment. This allows the model to find high-level design features such as shape, contour, and structure while modeling long-range dependencies across different parts of a garment. The addition of these annotated landmarks significantly enhances the model’s competency in detecting important garment areas that are needed to generate critical patterns, including collars, hemlines, and seams. In this respect, transformer-based models process the description of the garment if available for text encoding.

BERT is the state-of-the-art NLP model that understands context from a new perspective: it scans text in both directions, forward and reverse. Such a bidirectional process helps BERT catch the subtle shades of meaning and relations among words, providing deep semantic insight (Girawan and Alamsyah, 2023). This study uses BERT to derive information regarding style, material, and design intent that enriches the interpretation of textual data, completing the visual input. It then fuses them in a multi-modal fusion mechanism after feature extraction in both vision and text. This approach exploits cross-attention in aligning the effective mergers of information contributed by both modalities into a single coherent representation of a garment design. For example, visual features about the structural aspects of a garment could provide a pair with the semantic cues of its textual description in such a way that both esthetic and functional features would be properly represented. With such advanced encoding and fusion methods, feature extraction will have the model grasp a solid understanding of the input design, thus creating this integrated representation that later will serve as the basis for generating patterns of fashion-precise and fitting within the context in further steps of the workflow.

### 3.3 Multi-modal architecture and training strategy

The proposed framework employs a multi-modal Deep Learning architecture that integrates visual garment analysis, textual semantic understanding, and generative pattern synthesis within a unified end-to-end workflow. The system architecture consists of four major modules: visual feature extraction, textual feature encoding, multi-modal feature fusion, and GAN-based pattern generation.

For visual feature extraction, garment images resized to 256 × 256 pixels were processed using a Vision Transformer (ViT) architecture. Each image was divided into fixed-size patches of 16 × 16 pixels and projected into embedding vectors combined with positional encodings to preserve spatial relationships among garment components. The transformer encoder consisted of 12 layers with multi-head self-attention mechanisms that enabled the model to capture global structural dependencies between garment regions such as collars, sleeves, cuffs, and hemlines.

Textual garment descriptions were encoded using Bidirectional Encoder Representations from Transformers (BERT). Garment attributes including neckline type, sleeve style, fabric category, and fit description were tokenized and transformed into contextual semantic embeddings using the final hidden representation generated by the BERT encoder. This process enabled semantic interpretation of garment construction requirements and stylistic characteristics.

Following feature extraction, a cross-modal attention fusion mechanism was employed to combine visual and textual embeddings into a unified latent representation. The fusion process aligned semantic garment attributes with corresponding visual structures to improve pattern consistency, landmark accuracy, and manufacturing-oriented structural consistency.

The fused latent representation was subsequently passed into an encoder–decoder Generative Adversarial Network (GAN) architecture for pattern generation and refinement. The generator network consisted of convolutional upsampling layers that transformed latent embeddings into garment pattern maps, while the discriminator network evaluated pattern realism, seam continuity, structural consistency, and manufacturability quality. Through adversarial training, the generator iteratively improved pattern outputs based on discriminator feedback to progressively improve the structural consistency and visual plausibility of the generated pattern representations.

The training process was conducted using the Adam optimizer with a learning rate of 1 × 10^−4^, batch size of 32, and 100 training epochs. Three complementary loss functions were employed during optimization. Cross-Entropy Loss was used for garment attribute classification, Mean Squared Error (MSE) Loss was applied to minimize landmark alignment errors, and Adversarial Loss was utilized to improve the realism and structural quality of generated patterns.

To ensure experimental reproducibility and fair comparison, all baseline models were trained using identical dataset partitions, preprocessing procedures, augmentation strategies, hardware configurations, and optimization parameters. The experiments were implemented using PyTorch and TensorFlow frameworks on NVIDIA A100 GPUs with 128 GB RAM.

Model training was performed for 100 epochs using a batch size of 32 and the Adam optimization algorithm with an initial learning rate of 1 × 10^−4^. During each training iteration, visual and textual representations were extracted using the ViT and BERT encoders, respectively, and subsequently integrated through the cross-modal attention mechanism. The resulting fused representation was supplied to the encoder–decoder generator to produce the initial pattern prediction. The discriminator then evaluated the generated output relative to the reference representation, providing adversarial feedback for iterative optimization. Cross-Entropy Loss was employed for garment attribute-related prediction, Mean Squared Error was used to minimize deviations between predicted and reference landmark positions, and Adversarial Loss guided the generator toward structurally realistic pattern outputs. Validation performance was monitored separately from the training procedure to assess convergence and generalization.

For comparative reproducibility, the proposed framework and both baseline architectures were evaluated using the same dataset partitions and input resolution. Identical preprocessing and augmentation procedures were maintained wherever applicable, and the principal optimization settings, including the number of epochs, batch size, and learning rate, were held constant across comparative experiments. This controlled experimental design was adopted to minimize the influence of external training variations and to ensure that observed performance differences primarily reflected differences among the evaluated model architectures.

**Figure 3:**
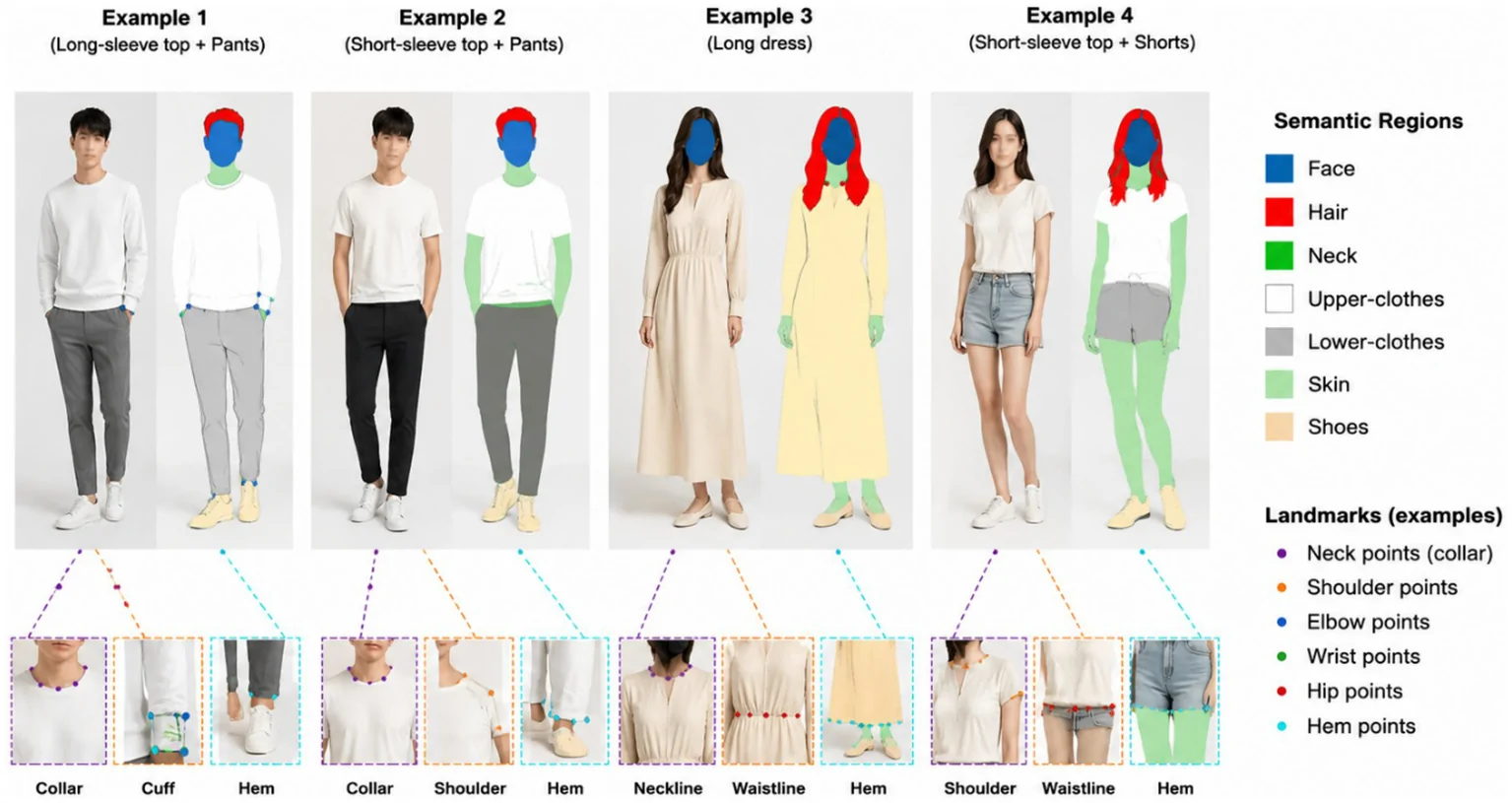
Proposed multi-modal deep learning framework for automated generation of CAD-compatible garment pattern representations, integrating ViT-based visual feature extraction, BERT-based textual encoding, cross-modal attention fusion, encoder–decoder GAN pattern generation and refinement, construction-detail integration, CAD export, and CLO3D-based virtual assessment. Images taken from dataset: https://www.kaggle.com/datasets/vishalbsadanand/deepfashion-1/code.

To provide a clearer representation of the proposed framework, Figure 3 illustrates the complete multi-modal Deep Learning architecture developed for automated fashion pattern generation. The framework integrates visual garment analysis, textual semantic encoding, cross-modal feature fusion, GAN-based pattern synthesis, manufacturability optimization, and CAD-oriented validation within a unified end-to-end pipeline. The figure demonstrates the sequential flow of information from garment input acquisition to final CAD-compatible pattern-representation generation.

As illustrated in Figure 3, the proposed framework combines transformer-based visual and textual feature extraction with cross-modal fusion and adversarial pattern refinement to generate structurally consistent garment pattern representations for downstream CAD processing and virtual assessment. The integration of ViT, BERT, cross-modal attention, and GAN-based refinement enables the framework to preserve garment-related semantic and structural information, improve landmark alignment and geometric consistency, and support subsequent CAD export and CLO3D-based virtual garment assessment.

### 3.4 Pattern generation

Pattern generation represents the core stage of the automated fashion pattern creation workflow, where advanced generative models, including GANs and transformer-based architectures, generate detailed and structurally consistent garment pattern representations. These models address the complexities of garment design by producing structurally accurate and esthetically consistent patterns that satisfy both functional and visual requirements. The pattern generation process consists of four major stages: initial pattern drafting, GAN-based refinement, conditional attribute guidance, and manufacturability optimization.

#### 3.4.1 Initial pattern drafting

Initial pattern drafting begins by combining the encoded visual and textual features through an encoder–decoder architecture. The encoder compresses the extracted multi-modal features into a compact latent representation that captures the structural and semantic characteristics of the garment design, including sleeve length, neckline style, silhouette, and fabric properties. This latent representation is subsequently passed into the decoder network, which reconstructs an initial technical garment pattern.

The generated draft serves as the primary structural blueprint of the garment and includes critical design elements such as seam lines, darts, notches, and contour boundaries. Garment landmarks including collars, cuffs, armholes, and hemlines guide the geometric construction of the initial pattern. For example, long-sleeved garments include refined armhole contours and cuff structures, whereas fitted garments incorporate shaping elements and dart placements to achieve proper silhouette alignment. This stage ensures that the generated pattern preserves both the esthetic characteristics and structural integrity of the original garment design.

#### 3.4.2 GAN-based pattern refinement

Following initial drafting, the generated pattern undergoes an iterative refinement process using a Generative Adversarial Network (GAN). The refinement stage improves structural consistency, geometric coherence, and visual realism, and visual realism by progressively optimizing the generated pattern output.

The generator network continuously refines pattern boundaries, seam continuity, edge smoothness, and structural alignment, while the discriminator network evaluates the realism and integrity of the generated garment pattern. Through adversarial feedback, the discriminator identifies inconsistencies or unrealistic structural characteristics and guides the generator toward producing more accurate and structurally coherent outputs.

This adversarial refinement process reduces visual artifacts and improves seam alignment and geometric consistency relative to the reference representations. As a result, the generated pattern representations exhibit progressively improved contour and structural consistency.

#### 3.4.3 Conditional attribute guidance

Conditional generation mechanisms are incorporated to ensure that the generated garment patterns remain consistent with predefined design specifications and garment attributes. These attributes include garment category, fabric behavior, fit characteristics, sleeve configuration, and silhouette requirements.

For instance, fitted garments such as tailored blazers require structured lapel shaping and silhouette refinement, whereas casual garments such as hoodies prioritize loose-fitting structures and simplified contour configurations. The conditional guidance mechanism also incorporates fabric-specific properties such as rigidity, elasticity, and draping behavior to adapt the generated patterns according to material characteristics.

This conditioning strategy improves the semantic alignment between input garment descriptions and generated technical patterns while enhancing design controllability and structural consistency.

#### 3.4.4 Manufacturability optimization

The final stage focuses on optimizing the generated patterns for industrial garment manufacturing applications. Production-oriented elements including seam allowances, grainlines, notch placements, and assembly markers are incorporated into the generated patterns to ensure compatibility with standard garment construction workflows.

The optimization process also improves pattern usability by refining geometric consistency, smoothing irregular contours, and preserving proportional garment relationships. Through iterative refinement and structural validation, the framework produces pattern representations with improved geometric consistency for downstream CAD and virtual garment workflows.

By integrating multi-modal feature extraction, adversarial refinement, conditional guidance, and manufacturability optimization within a unified workflow, the proposed framework provides a scalable and production-oriented solution for automated fashion pattern generation.

It should be emphasized that the manufacturability optimization performed in the present study represents a computational and virtual pre-production stage. The incorporation of seam allowances, grainlines, notch placements, and assembly markers prepares the generated pattern representations for downstream CAD and virtual garment assessment; however, the presence of these construction elements does not by itself establish physical manufacturability. Definitive validation would require the generated patterns to be cut from fabric, physically assembled, and evaluated against professional garment-construction criteria. Accordingly, the present framework assesses manufacturing-oriented pattern characteristics and virtual assembly feasibility rather than experimentally verified physical manufacturability.

### 3.5 Validation and post-processing

After the pattern generation step, the patterns have to be evaluated for geometric accuracy and visual consistency before downstream CAD and virtual garment assessment. The process is very critical as it makes the patterns esthetically and pragmatically appropriate to the original intention and requirements of garment manufacturing. Validation considers a few key metrics, such as the quality or reliability of the mined patterns.

#### 3.5.1 Alignment of landmarks

This shows how much the landmark patterns have been aligned, taking into consideration how well those patterns fit the predefined garment landmarks like collars, cuffs, and hemlines that ensure the structural integrity of the pattern.

#### 3.5.2 Esthetic consistency

It essentially checks whether the generated patterns correspond to the input design visually in every aspect, be it contours, proportions, or even stylistic details.

#### 3.5.3 Intersection over union

This will give, in quantitative terms, how much overlap generated patterns have against the pseudo-pattern labels as an indication of precision. After validation, these patterns move to the post-processing stage, where they will be fine-tuned to meet the standards of real-world usability.

#### 3.5.4 Smoothening and cleaning

It deals with every irregularity or artifact of the generated patterns, such as smoothening the curve and making the lines uniform, which is imperative for professional output.

#### 3.5.5 Adding production details

Involves adding critical manufacturing information to the designs, such as seam allowances, grainlines, and notches. These are critical when it comes to fabric cutting and garment assembly, preparing the pattern representations for subsequent CAD processing and virtual assembly assessment. Including these steps of validation and post-processing while building the system will ensure that the final pattern representations are geometrically refined, visually consistent, and prepared for downstream CAD and virtual garment evaluation. Such a holistic approach fills the gap between automatically generated patterns and their real-world applicability, turning the system into a valued component in the workflow of modern fashion design.

## 4 Integration with CAD systems

These generated patterns then integrate with computer-aided design systems for the practicality and usability of garment production workflow. Translating the patterns into industrially accepted vector formats, such as SVG and DXF, which can then be easily read by digital cutting machines or CAD software, popularly deployed in the fashion industry. These formats help maintain the patterns with critical manufacturing information like seam allowances, grainlines, and notches, which are important during the processes of accurate fabric cutting and assembly. This allows scaling and modifications with much ease. The designer and manufacturer will be able to change the patterns for certain size ranges or variations of a garment without any loss of accuracy. More information, such as alignment markers and specifications of fabric types, can also be added as metadata within these vector files to assist the production process further. Optimized with digital workflows in mind, these enable seamless transitions from design to downstream virtual garment development. Afterward, the patterns are checked in CLO3D virtual 3D garment simulation software. CLO3D is a powerful 3D garment simulation software widely used in the fashion and apparel industry. Pattern simulation is an important tool for visualizing how garments look and behave in real life. Properties that were analyzed during the simulation are given in Table 1.

**Table 1:** Simulation properties.

Property	Description
Fit	The pattern needs to ensure the garment pieces form an actual-to-size garment.
Draping	Assessing how fabrics fall and contour around a 3D model based on material properties.
Assembly	This involves verifying alignment and compatibility in garment construction through pattern pieces.

### 4.1 CLO3D-based validation procedure

CLO3D-based virtual simulation was employed as the final validation stage to determine whether the generated two-dimensional patterns could be assembled into structurally coherent three-dimensional garments. Following pattern generation and post-processing, the pattern pieces were converted into CAD-compatible vector representations while preserving panel boundaries, seam information, notches, grainlines, and other available construction markers. The generated pattern pieces were subsequently imported into the CLO3D environment and positioned around the corresponding virtual avatar according to their intended garment locations.

Before simulation, the sewing relationships between adjoining pattern edges were defined according to the generated seam information. Pattern orientation and grainline direction were checked to ensure appropriate positioning, and the required fabric properties were assigned within the virtual environment. The garment pieces were then virtually assembled and simulated on the avatar until a stable draped configuration was obtained. This procedure enabled the two-dimensional pattern geometry to be examined under three-dimensional garment assembly conditions rather than relying exclusively on image-based pattern similarity.

Validation was performed across three principal dimensions: fit, draping, and assembly compatibility. Fit assessment examined whether the assembled pattern produced the intended garment proportions and whether major garment regions were appropriately positioned relative to the avatar. Particular attention was given to neckline placement, shoulder positioning, sleeve length, garment length, and overall silhouette. Draping assessment examined whether the generated pattern produced visually coherent fabric behavior over the three-dimensional body surface without substantial geometric distortion. Assembly validation focused on the compatibility of adjoining pattern pieces, including correspondence between seam lengths, alignment of connected edges, sleeve-to-armhole relationships, and continuity between major garment panels.

The simulation was additionally inspected for visible indicators of pattern-generation errors, including seam misalignment, unintended openings, excessive distortion, abnormal fabric tension, overlapping pattern pieces, and inconsistent garment symmetry. When such problems were detected, the corresponding generated pattern was identified as requiring refinement rather than being considered directly production-ready. This simulation-based inspection therefore provided an additional manufacturability check complementary to the quantitative IoU and landmark-alignment metrics.

The CLO3D validation stage was not intended to replace physical prototype testing; rather, it served as a virtual pre-production assessment of pattern plausibility and assembly compatibility. Combining quantitative two-dimensional pattern evaluation with three-dimensional garment simulation provided a more comprehensive assessment of whether the generated outputs retained both geometric accuracy and practical garment-construction characteristics.

These simulations will enable the location of misaligned seams, poor fits, or other fabric tension problems that can be resolved early without having to address expensive mistakes in physical prototyping.

**Figure 4:**
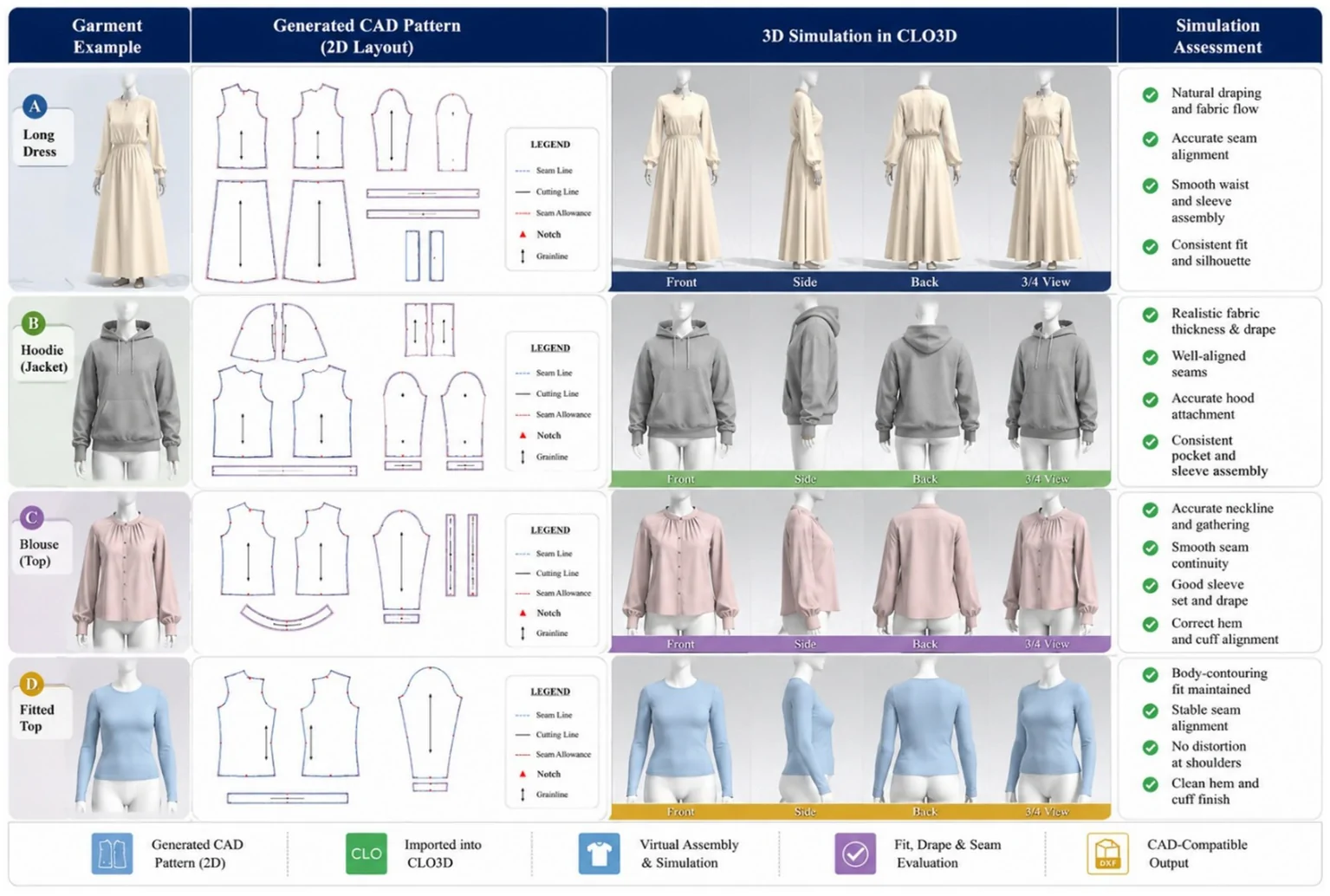
CLO3D-based virtual assessment of generated CAD-compatible garment pattern representations across multiple garment categories, showing the relationship between two-dimensional pattern layouts and corresponding three-dimensional simulations for evaluating fit, draping, seam alignment, and assembly compatibility. Images taken from dataset: https://www.kaggle.com/datasets/vishalbsadanand/deepfashion-1/code.

To further evaluate the virtual assembly feasibility and structural behavior, Figure 4 presents representative CLO3D-based validation results. The figure demonstrates the relationship between the generated technical garment patterns and their corresponding three-dimensional garment simulations under virtual production conditions. The visualization enables assessment of garment draping behavior, seam alignment accuracy, fitting consistency, and structural assembly compatibility.

As illustrated in Figure 4, the generated garment patterns maintained strong structural consistency and manufacturability during CLO3D simulation. The simulated garments demonstrated accurate seam alignment, realistic fabric draping behavior, stable assembly compatibility, and satisfactory fitting performance across multiple garment categories. These results demonstrate that the generated pattern representations can be transferred to a CAD-supported virtual garment workflow and assessed for structural consistency, seam relationships, fit, and draping in CLO3D. The findings support the feasibility of the proposed framework as a preliminary digital pattern-generation approach; however, industrial production readiness cannot be established without comparison against professionally drafted CAD patterns and physical garment validation. Further, this also allows the designer to test different fabric types or variations in design virtually with great efficiency and thus perform rapid iteration and refinement. This would not only make generated patterns available in CAD systems or 3D simulation tools but also simplify garment production by improving levels of accuracy, reducing waste, enhancing time-to-market, and bridging the gap from design automation to manufacturing. This virtual workflow may support earlier identification of structural issues and more efficient iterative refinement before physical prototyping.

The CLO3D assessment should therefore be interpreted as a virtual pre-production feasibility evaluation rather than definitive validation of physical manufacturability. Successful simulation indicates that the generated pattern representations can be assembled virtually and examined for fit, draping, seam relationships, and gross structural inconsistencies, but it does not demonstrate that the same patterns can be cut, sewn, finished, and assembled successfully under physical production conditions. Factors such as fabric handling during cutting and sewing, seam construction tolerances, ease requirements, material deformation, sewing-machine constraints, and finishing operations cannot be fully established through virtual simulation alone. Physical prototype construction from the generated patterns is therefore required before claims of industrial manufacturability can be confirmed.

## 5 Experimental setup and results

The experimental framework was executed on the workstation with NVIDIA A100 GPUs and 128GB RAM to ensure high-performance computation for model training and inference. PyTorch and TensorFlow were utilized to develop the models due to their robust libraries in advanced Deep Learning tasks. The training was done with 100 epochs, a batch size of 32, and a learning rate of 1 × 10^−4^. In the model, three loss functions were used: Cross-Entropy Loss for classification tasks, Mean Squared Error to minimize pattern alignment error, and Adversarial Loss for refining the generation of patterns within the GAN framework.

The selected metrics evaluate the geometric accuracy, visual consistency, and virtual feasibility of the generated pattern representations. IoU calculates the spatial overlap between the generated pattern representations and the corresponding DeepFashion-derived pseudo-pattern references. The Landmark Alignment Error calculates the positional accuracy of garment-specific landmarks, such as collars and hemlines, while Esthetic Consistency assesses the visual fidelity of generated patterns to the input design through expert reviewing. These provide a comprehensive handle on system performance.

The Intersection over Union (IoU) metric was computed by measuring the overlap ratio between generated garment pattern regions and corresponding pseudo-pattern reference labels. Landmark Alignment Error was calculated as the average Euclidean pixel distance between predicted garment landmarks and annotated ground truth landmark positions. Esthetic Consistency was evaluated through expert assessment conducted by five professional fashion designers independently using a 10-point Likert evaluation scale based on visual coherence, structural realism, and similarity to the original garment design. Pattern Accuracy was determined by measuring the percentage of correctly generated structural pattern components relative to the reference patterns. Scalability Efficiency and Expert Usability Score were evaluated based on system responsiveness, workflow integration capability, and practical usability within CAD-supported garment production environments.

### 5.1 Evaluation procedure

The evaluation procedure was designed to assess the generated patterns from complementary geometric, structural, visual, computational, and practical perspectives. All quantitative evaluations were conducted on the held-out test subset, which was not used for model parameter optimization. The same test samples and evaluation criteria were applied to the proposed framework and baseline models to ensure direct comparability.

Intersection over Union (IoU) was used to quantify the spatial correspondence between the generated pattern region and its corresponding pseudo-pattern reference. For each test sample, IoU was calculated as the area of intersection between the predicted and reference pattern regions divided by their area of union. Values closer to 1 indicate greater spatial agreement. Landmark Alignment Error was calculated as the Euclidean distance, measured in pixels, between predicted and reference garment landmarks, including structurally important locations such as neckline points, sleeve boundaries, armholes, cuffs, and hemlines. The reported value represents the average landmark error across the evaluated samples, with lower values indicating greater structural precision.

Pattern Accuracy was assessed by determining the proportion of reference structural pattern components that were correctly represented in the generated output. In addition to these quantitative measures, esthetic Consistency was evaluated independently by five professional fashion designers using a 10-point rating scale. The experts considered visual coherence, proportional consistency, structural realism, and correspondence between the generated pattern and the original garment design. Expert Usability Score additionally assessed the practical suitability of generated outputs for patternmaking and CAD-supported garment development.

Manufacturability was further examined through CLO3D-based virtual garment simulation. Generated patterns were imported into the virtual environment and assessed in terms of fit, fabric draping, seam and pattern-piece alignment, and assembly compatibility. This stage was used to identify structural problems that may not be fully captured by image-based quantitative metrics alone, including incompatible adjoining edges, distorted assembly, or problematic garment fit. Pattern Refinement Time was recorded to assess computational efficiency, while Scalability Efficiency reflected the system’s ability to maintain practical workflow performance during repeated pattern-generation operations. Together, these evaluation procedures provided a multi-dimensional assessment of geometric accuracy, visual fidelity, computational performance, and manufacturing applicability.

There were multiple stages to the evaluation pipeline. Input data in the form of garment images, sketches, and attributes passed through common visual and textual encoders to extract unified features. These are then passed through to an encoder–decoder network that outputs initial patterns, which are further refined using GANs. During post-processing, the patterns became smooth, and critical details like seam allowance and grainline were added, making them manufacturable. The generated patterns were finally tested in CLO3D, a 3D garment simulation tool, to validate their fit, draping, and assembly under realistic conditions.

**Figure 5:**
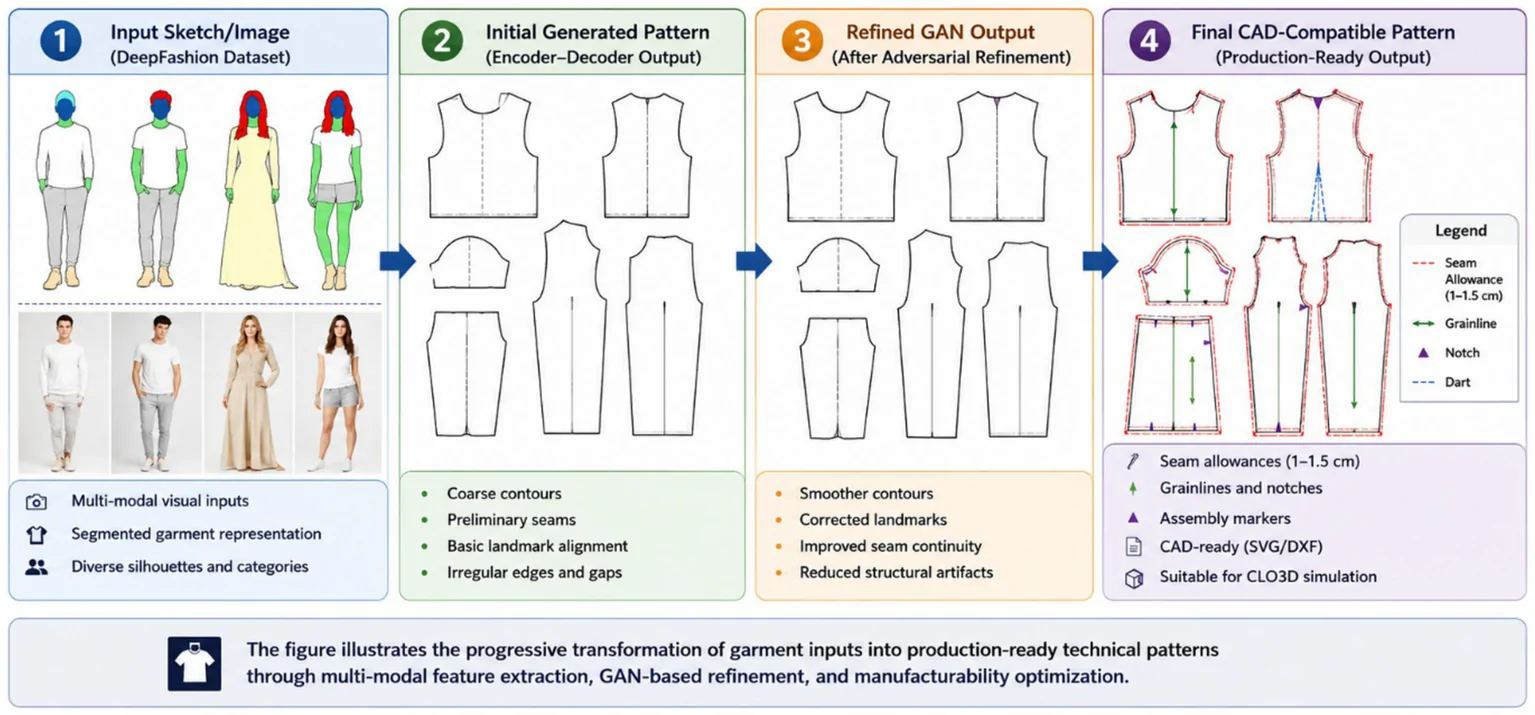
Intermediate stages of automated fashion pattern generation. Images taken from dataset: https://www.kaggle.com/datasets/vishalbsadanand/deepfashion-1/code.

To further illustrate the effectiveness of the proposed framework, Figure 5 presents the intermediate stages of automated fashion pattern generation. The figure demonstrates the progressive transformation of garment inputs into production-ready CAD-compatible patterns through encoder–decoder generation, GAN-based refinement, and manufacturability optimization. These qualitative results provide visual evidence of improvements in contour smoothness, seam continuity, landmark alignment, and structural consistency throughout the generation pipeline.

The proposed framework was evaluated against two baseline configurations implemented under identical experimental conditions to ensure fair performance comparison. Baseline 1 consisted of a conventional Convolutional Neural Network (CNN)-based architecture that performed garment feature extraction and direct pattern generation without transformer-based contextual modeling, multi-modal fusion, or adversarial refinement mechanisms. Baseline 2 employed a conditional Generative Adversarial Network (cGAN) architecture for garment pattern generation but excluded the proposed cross-modal attention fusion strategy, landmark-guided structural alignment, and automated seam and notch generation modules.

All models were trained using the same DeepFashion dataset partitions, identical preprocessing procedures, augmentation strategies, optimization parameters, hardware configurations, and evaluation metrics. This standardized experimental setup ensured that performance differences resulted from architectural improvements rather than variations in training conditions or dataset composition.

The experimental results and comparative performance evaluations are presented in Tables 2, 3.

**Table 2:** Performance comparison of the proposed system with baseline models.

Metric	Proposed system	Baseline 1 (CNN)	Baseline 2 (cGAN)
IoU	0.93	0.81	0.87
Landmark alignment error	3.2 px	8.5 px	5.4 px
Esthetic consistency	9.5/10	7.2/10	8.3/10
Pattern accuracy (%)	96.2%	85.6%	91.3%
Pattern refinement time (s)	0.5 s	0.9 s	0.7 s
Scalability efficiency	98%	85%	90%
Expert usability score	92%	70%	85%

**Table 3:** Methodological comparison with recent digital garment and sewing-pattern generation approaches.

Approach	Primary task	Representation/method	Sewing-pattern output	Multimodal input	Pattern-level ground truth	Virtual/3D validation
DressCode	Multi-category virtual try-on	Garment-conditioned image generation	No	Limited	No	No
SewFormer	Sewing-pattern reconstruction	Hierarchical Transformer	Yes	No	Yes	Yes
GarmentCode	Parametric garment construction	Programmatic/parametric pattern representation	Yes	Parameter-driven	Procedurally defined	Yes
Recent diffusion-based approaches	Garment/pattern generation	Diffusion-based generative modeling	Method-dependent	Yes	Method-dependent	Method-dependent
Proposed framework	Multimodal pattern-representation generation	ViT + BERT + cross-modal fusion + GAN	**Yes, pseudo-pattern based**	**Yes**	**No—DeepFashion-derived pseudo-patterns**	**CLO3D virtual assessment**

Bold text indicates the distinctive or enhanced capabilities of the proposed framework compared with the other approaches, particularly its pseudo-pattern-based sewing-pattern output and CLO3D virtual assessment.

**Figure 6:**
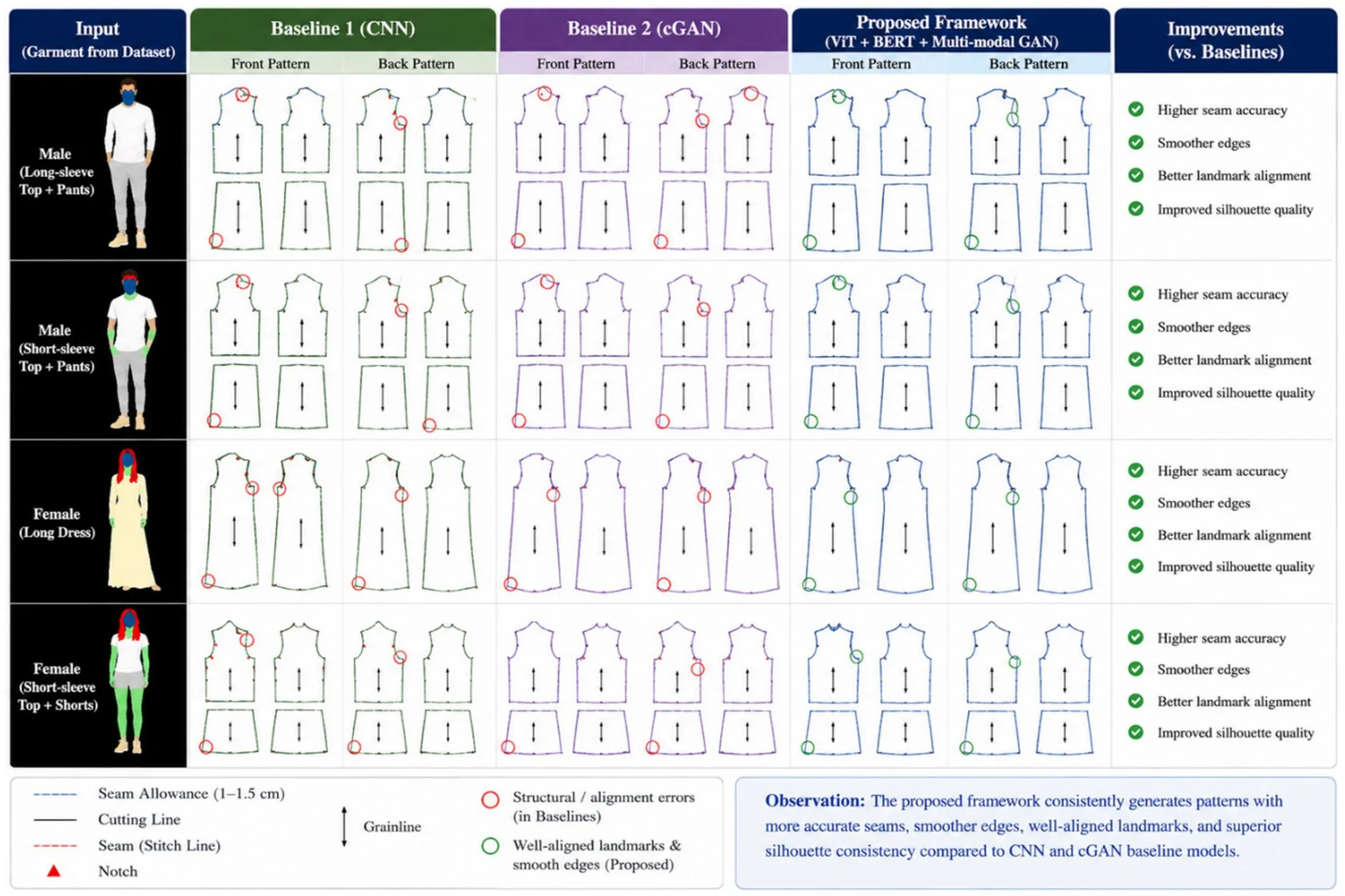
Visual comparison between the proposed framework and baseline models. Images taken from dataset: https://www.kaggle.com/datasets/vishalbsadanand/deepfashion-1/code.

To further validate the effectiveness of the proposed framework, Figure 6 presents a qualitative visual comparison between the proposed system and baseline CNN and cGAN models. The figure illustrates differences in seam accuracy, edge smoothness, landmark alignment, and overall garment silhouette quality across multiple garment samples extracted from the DeepFashion dataset.

As illustrated in Figure 6, the proposed framework generates garment patterns with smoother contours, improved structural consistency, more accurate seam alignment, and better preservation of garment silhouettes compared with the CNN and cGAN baseline models. The visual comparison further confirms the quantitative improvements observed in IoU scores, landmark alignment accuracy, and esthetic consistency evaluations.

Recent specialized garment-generation approaches provide more technically relevant reference points than conventional CNN and cGAN baselines alone. Therefore, the comparative analysis was expanded to include DressCode, SewFormer, GarmentCode, and recent diffusion-based garment-generation approaches. These methods represent different but complementary developments in digital fashion research. DressCode addresses high-resolution multi-category virtual try-on and provides an important benchmark for garment-conditioned image synthesis, whereas SewFormer directly addresses sewing-pattern reconstruction from a single garment image using a hierarchical Transformer architecture and ground-truth sewing-pattern supervision. GarmentCode approaches the problem from a parametric perspective by representing valid sewing patterns through a domain-specific programming framework, thereby supporting structured garment construction and design manipulation. SewingLDM (Liu et al., 2025) further demonstrate the potential of generative models for multimodal and controllable garment and sewing-pattern generation.

Because these approaches differ substantially in task definition, datasets, garment representations, and evaluation protocols, their published numerical results are not directly comparable with the IoU and landmark-error values obtained using the DeepFashion-derived evaluation protocol adopted in the present study. Accordingly, the expanded comparison is presented at the methodological and capability levels rather than treating results obtained from different datasets as directly equivalent quantitative benchmarks. This distinction is particularly important for specialized methods trained on datasets containing actual sewing-pattern ground truth, which provide a stronger basis for pattern-level evaluation than image-landmark datasets.

The expanded comparison highlights an important distinction between the proposed framework and specialized sewing-pattern methods. SewFormer benefits from SewFactory, which contains explicit sewing-pattern ground truth and stitching information, making it particularly suitable for quantitative pattern-reconstruction evaluation. GarmentCode, in contrast, ensures structural validity through a programmatic and parametric garment representation. These approaches therefore provide stronger pattern-specific references than conventional image-generation baselines. The proposed framework differs in its emphasis on combining visual and textual garment information with downstream CAD-oriented processing and CLO3D assessment; however, its current DeepFashion-based supervision does not provide equivalent industrial sewing-pattern ground truth. Consequently, direct quantitative comparison with specialized sewing-pattern systems should be conducted in future work using a shared dataset and common pattern-level metrics. Such benchmarking would enable a more rigorous assessment of panel geometry, stitching relationships, structural validity, and simulation compatibility across competing methods.

The proposed system achieved the highest Intersection over Union (IoU) score of 0.93 and the lowest landmark alignment error of 3.2 pixels, indicating improved structural precision and pattern consistency. Furthermore, unlike several existing approaches, the proposed framework incorporates both multi-modal feature fusion and CLO3D-based simulation validation, enabling direct assessment of garment fit, draping behavior, and assembly compatibility under realistic manufacturing conditions. “These results demonstrate the effectiveness of combining transformer-based feature extraction with adversarial refinement for structured garment pattern-representation generation under the adopted DeepFashion-derived evaluation protocol.

It is clear from the IoU score of 0.93 that the newly proposed system was able to detect greater spatial correspondence with the pseudo-pattern references higher than those of 0.81 and 0.87, which were lower than the baselines. The Landmark Alignment Error was significantly less at 3.2 pixels, which confirms that the placement of garment-specific landmarks was indicating improved landmark localization accuracy, while the baselines made unintentional errors of 8.5 px and 5.4 px. Thanks to a 9.5/10 score, which is the highest, the experts fully endorsed the system’s Esthetic Consistency for having closely replicated the input design’s visual elements, which were rated at 7.2/10 and 8.3/10 for the other two competition results.

The proposed system is only ahead of Pattern Accuracy with 96.2% compared to the baselines’ 85.6% and 91.3%, respectively. Less than half a second for the Pattern Refinement Time is a good indication of the system’s time reduction. Hence, the system is really seamless and works for real-time applications; it has to have a Scalability Efficiency of 98%. The Expert Usability Score of 92% shows a high level of satisfaction with the patterns’ quality and usability, compared to the scores of 70% and 85% for the baselines.

The observed performance of the complete framework is consistent with the intended complementary roles of its visual, textual, and generative components. ViT provides a mechanism for modeling global relationships among garment regions, while BERT supplies semantic representations of textual garment attributes, and cross-modal attention integrates information from the two modalities before pattern generation. The GAN-based refinement stage subsequently operates on the fused representation to improve the generated output. However, because these components were evaluated as part of the integrated architecture rather than through controlled component-removal experiments, the present results do not establish the independent quantitative contribution of ViT, BERT, or cross-modal fusion. A dedicated ablation analysis is therefore required to determine how much each component contributes to the overall performance.

The proposed Deep Learning framework demonstrates the feasibility of automatically generating structured garment pattern representations with strong geometric correspondence and compatibility with downstream CAD and virtual garment-simulation workflows. The results support the potential of combining multi-modal feature extraction, transformer-based representation learning, and generative refinement for automated fashion pattern development. However, the present evaluation establishes geometric accuracy and virtual assembly feasibility rather than physical manufacturability. Physical garment construction and professional patternmaking assessment remain necessary before the generated patterns can be considered validated for industrial production.

These results arise from the combination of multi-modal feature extraction based on Vision Transformers for images and transformer-based language models for text, further combined with iterative refinement using GANs, yielding pattern representations with improved geometric and visual consistency. Besides these remarkable results, this paper demonstrates the usability of Deep Learning for automating what is traditionally a very time-consuming, specialized task. This framework bridges this gap by first integrating important pattern information like seam allowances and grain lines and then testing their interactions via 3D simulations. By smooth integration, it will reduce the likelihood of errors, quicken the development cycle, and make the skills of pattern-making more accessible to the common people. These are some of the positive observations for further studies. This automation pipeline would be further reinforced by expanding the framework to other garment categories, using body-scan data for personal fits, and including further constraints such as sustainability goals or adaptive fabric properties. In general, this work points out how Deep Learning based automation is going to reshape the fashion sector, opening new horizons toward innovative, efficient, and scalable design solutions.

## 6 Conclusion

This study presented a multi-modal Deep Learning framework for automated generation of structured garment pattern representations from visual and textual design information. The framework integrates ViT-based visual encoding, BERT-based textual representation, cross-modal feature fusion, and GAN-based generative refinement, followed by CAD-oriented post-processing and CLO3D-based virtual assessment. Under the DeepFashion-derived evaluation protocol, the proposed framework achieved an IoU of 0.93 and an average landmark alignment error of 3.2 pixels, demonstrating strong geometric correspondence with the pseudo-pattern references.

The findings demonstrate the potential of multi-modal Deep Learning to support automated digital pattern-development workflows and integration with CAD and three-dimensional garment simulation environments. However, the present results establish geometric correspondence and virtual assembly feasibility rather than physical manufacturability or industrial production readiness. DeepFashion does not provide professionally drafted sewing-pattern ground truth, and physical garments were not constructed from the generated outputs. Future research should therefore evaluate the framework using paired image–CAD pattern datasets, controlled component-level ablation experiments, blinded professional patternmaker assessments, and physical garment prototypes. These extensions would provide a stronger basis for evaluating the practical applicability of AI-generated garment patterns in professional and industrial fashion workflows.

## 7 Limitations

A principal limitation of this study concerns the available ground-truth data. Although DeepFashion provides a large and diverse collection of garment images, attributes, and landmark annotations, it does not provide professionally drafted sewing patterns with verified seam allowances, grainlines, notches, grading rules, stitching relationships, or construction specifications. The pseudo-pattern references used in this study therefore support the evaluation of geometric correspondence and landmark consistency but cannot serve as industrial ground truth for validating production-ready garment patterns. Future research should evaluate the framework using paired datasets containing garment images and professionally drafted CAD patterns to enable more rigorous pattern-level validation.

A second limitation concerns physical manufacturability. CLO3D simulation provides useful virtual evidence regarding pattern assembly, fit, draping, and structural consistency, but it cannot replace physical prototype construction and professional patternmaker assessment. Future experiments should therefore export generated patterns to CAD-compatible formats, cut and sew physical garment prototypes, and evaluate them for seam compatibility, dimensional accuracy, ease, balance, fit, assembly quality, and construction defects. Comparing physical prototypes with both the generated pattern specifications and CLO3D simulations would provide stronger evidence regarding the applicability of the framework to actual garment production.

A further limitation is the absence of a controlled component-level ablation study. The proposed architecture integrates ViT-based visual encoding, BERT-based textual encoding, cross-modal attention fusion, and GAN-based refinement; however, their individual contributions were not isolated experimentally. Consequently, the reported results demonstrate the performance of the integrated framework and should not be interpreted as evidence of the independent contribution of any individual component. Future work should compare the complete framework with controlled variants that remove or replace the BERT branch, ViT encoder, cross-modal attention mechanism, and GAN refinement stage while maintaining identical dataset partitions, training conditions, and evaluation criteria. Such experiments would enable the contribution of each architectural component to be quantified more rigorously.

## Data Availability

The original contributions presented in the study are included in the article/supplementary material, further inquiries can be directed to the corresponding author/s. The implementation developed for this study is not currently publicly available. The manuscript provides the model architecture, preprocessing procedure, principal training parameters, and evaluation methodology to support methodological transparency. Future work will consider releasing a reproducible implementation subject to institutional requirements and applicable dataset-use conditions.
